# E-cigarette and waterpipe use in two adolescent cohorts: cross-sectional and longitudinal associations with conventional cigarette smoking

**DOI:** 10.1007/s10654-017-0345-9

**Published:** 2017-12-19

**Authors:** Jorien L. Treur, Andrea D. Rozema, Jolanda J. P. Mathijssen, Hans van Oers, Jacqueline M. Vink

**Affiliations:** 1 Behavioural Science Institute, Radboud University Nijmegen, Nijmegen, The Netherlands; 2 Tranzo Scientific Center for Care and Welfare, Tilburg School of Social and Behavioral Sciences, Tilburg University, Tilburg, The Netherlands; 3 RIVM National Institute for Public Health and the Environment, Bilthoven, The Netherlands

**Keywords:** Adolescents, E-cigarettes, Waterpipe, Smoking, Longitudinal, Smoking propensity

## Abstract

Alternative tobacco products are increasing in popularity. An important question is whether their use is associated with or even leads to conventional smoking, but large-scale (European) studies are scarce. In two cohorts of Dutch adolescents (Cohort I n = 6819, mean age = 13.8 SD = 1.1, 48.2% female; Cohort II n = 2758, mean age = 17.3 SD = 1.8, 61.3% female), we investigated use of electronic (e)-cigarettes with nicotine, e-cigarettes without nicotine and waterpipe. Generalized estimating equation modelling was conducted with ever conventional smoking as the dependent variable (0 = no, 1 = yes) and ever alternative tobacco use as the independent variable, correcting for clustering within schools, age, sex and education in both cohorts. In a subsample (n = 2100), the association between alternative tobacco use at baseline and conventional smoking 6 months later was tested, taking into account smoking propensity (based on personality, susceptibility to peer pressure and smoking intentions). Ever use prevalence was 13.7% for e-cigarettes with nicotine, 29.4% for e-cigarettes without nicotine and 22.1% for waterpipe in Cohort I and 12.3, 27.6 and 45.3% respectively in Cohort II. Ever smokers had tried alternative tobacco products more often than never smokers. Among never-smoking adolescents at baseline, alternative tobacco use predicted ever smoking 6 months later (e-cigarettes with nicotine OR 11.90 95% CI 3.36–42.11; e-cigarettes without nicotine OR 5.36 95% CI 2.73–10.52; waterpipe OR 5.36 95% CI 2.78–10.31). This association was strongest for adolescents with a low baseline risk of smoking. Experimenting with alternative tobacco products is common among Dutch youth. Alternative tobacco use predicts (future) smoking, especially among adolescents with a low smoking propensity.

**Electronic supplementary material:**

The online version of this article (10.1007/s10654-017-0345-9) contains supplementary material, which is available to authorized users.

## Introduction

Alternative tobacco products are steadily increasing in popularity and are partly replacing ‘conventional’ cigarette smoking. Alternative tobacco products include electronic (e-)cigarettes with nicotine, e-cigarettes without nicotine (also known as ‘shisha-pens’) and waterpipe (also known as ‘shisha’ or ‘hookah’). A recent study from the United States (US) demonstrated that while adolescents’ use of conventional cigarettes was on the decline between 2011 and 2014, the net use of tobacco products remained the same due to the increasing popularity of alternative forms [1]. In 2013–2014, 40% of 45,971 adolescent and adult tobacco users from the US (aged ≥ 12 years) said they used multiple tobacco products with cigarettes and e-cigarettes being the most common combination [2]. E-cigarettes were originally intended as an aid for smoking cessation and although they are considered to be less harmful than conventional cigarettes [3], they are not risk free [4, 5]. Another important concern is that for individuals who never smoked before, e-cigarettes might form a ‘stepping stone’ to conventional cigarettes [6]. The same concern exists regarding waterpipe use [7], which in itself may be just as harmful as conventional smoking [8–10]. There is an increasing body of literature addressing the popularity of alternative tobacco products and its association with conventional cigarette smoking. Yet, large-scale European studies among adolescents and young adults, especially those exploring different types of alternative tobacco, are scarce.

The International Tobacco Control (ITC) Netherlands survey reported that in 2014, 40% of Dutch smokers aged 15 years or older had ever tried an e-cigarette and that 16% was currently using e-cigarettes [11]. In 2015, a national surveillance study among Dutch adolescents aged 12–16 years reported that the prevalence of ever using an e-cigarette, with or without nicotine, was 40% in boys and 29% in girls. This was considerably higher than the prevalence of ever using a conventional cigarette (24% in boys and 21% in girls). Of those who had used an e-cigarette, only 3% used the device weekly and 2% daily. When looking at adolescents who had used both a conventional cigarette and an e-cigarette, 35% stated to have tried the latter first [12]. The same study also assessed waterpipe, which had ever been used by 27% of boys and 18% of girls [12]. As with conventional smoking [13], sociodemographic factors are associated with the use of alternative tobacco products. For example, boys of 12–16 years were more prone to use alternative tobacco products compared to girls and, within this age group, a higher age was associated with an increased chance of ever having used an alternative tobacco product. In addition, a lower level of educational attainment was associated with using alternative tobacco products [12]. For more up to date numbers on the use of e-cigarettes and waterpipe and their sociodemographic patterning, not only in adolescents but also in young adults, more research is needed.

An important question is whether or not alternative tobacco products act as a stepping stone to conventional smoking. Among adolescents and young adults who had never smoked, e-cigarette use was associated with an increase in intention to smoke conventional cigarettes [14]. This may be because e-cigarettes renormalize conventional smoking by desensitizing adolescents to the dangers of smoking. Evidence for this was found by Miech et al. [15], who reported that using e-cigarettes decreases users’ perception of the (health) risk of conventional smoking [15]. However, it could also be that alternative tobacco products are simply a ‘precursor’ for other substance use, such that adolescents who use them would eventually also have started smoking conventional cigarettes. Longitudinal data can elucidate the temporal relationship of substance use behaviours and thereby shed some light on the causal nature of their relationship. A recent review of four longitudinal studies concluded that e-cigarette use was associated with an increased chance of using conventional cigarettes at a later time point, even in adolescents who were not considered to be ‘susceptible to smoking’ [6]. One of these studies suggested that e-cigarette use was associated with later smoking onset *especially* in adolescents who exhibited a low risk of smoking at baseline (lower levels of rebelliousness, willingness to smoke and higher levels of parental support) [16]. This was also found by Barrington-Trimis et al. [17], more recently [17]. Together, these findings suggest that alternative tobacco products act as a stepping stone to conventional cigarettes. However, most of the studies pertain US-based populations, no distinction has been made between e-cigarettes *with* and *without* nicotine before, and waterpipe use has not always been included.

In summary, the current literature is lacking non-US based, large-scale (longitudinal) studies that measure the use of e-cigarettes *with* and *without* nicotine, as well as waterpipe, and their association with conventional smoking. In addition, replication of previous findings that alternative tobacco use is associated with later conventional smoking more strongly in adolescents with a low propensity to smoke is crucial to strengthening evidence on whether or not alternative tobacco products act as a ‘gateway’ to conventional smoking. Therefore, in two large cohorts of Dutch adolescents from different age groups (Cohort I n = 6819 mean age = 13.8, Cohort II n = 2758 mean age = 17.3) we aimed to 1): investigate the prevalence and sociodemographic patterning of three major types of alternative tobacco (e-cigarettes with nicotine, e-cigarettes without nicotine and waterpipe) and 2): investigate the association between alternative tobacco and conventional smoking, cross-sectionally in the total sample and longitudinally in a subsample (n = 2100) whereby we take adolescents’ propensity to smoke, i.e. baseline smoking risk, into account. Based on the current knowledge, we hypothesized that alternative tobacco use would be associated with conventional smoking cross-sectionally and longitudinally. In addition, we expected longitudinal associations between alternative tobacco products and later conventional smoking to be stronger in adolescents that were considered to have a low baseline propensity to smoke.

## Methods

### Participants

Data on conventional cigarette smoking and the use of alternative tobacco products were available for two cohorts of Dutch adolescents. Cohort I consists of 6819 adolescents aged 11–17 years [mean age = 13.8 (SD = 1.1), 48.2% female] who were enrolled in a study that investigated the impact of school smoking policy on changes in adolescents’ smoking behaviour. Data were collected in 2014–2015 from 19 secondary schools randomly selected across the Netherlands [18]. A comprehensive description of this study is available in the supplementary material. Of the total of 6819 adolescents, 2100 had longitudinal data available on smoking and alternative tobacco use; at time point 0 (T0) and time point 1 (T1) with 6 months in between. At each time point, adolescents were asked to complete a survey containing questions on their smoking behaviour, personality and use of alternative tobacco products.

Cohort II consists of 2758 adolescent participants of the Tr&nds study (Traditional and Novel Substance use among Adolescents) aged 14 to 21 years [mean age = 17.3 (SD = 1.8), 61.3% female]. Tr&nds aims to assess addictive behaviour in a representative group of Dutch adolescents and young adults, with a particular focus on ‘novel’ types of addictive behaviour, including the use of alternative tobacco products [19]. Data were collected in 2016–2017 from 14 educational institutions located mostly in the West of the Netherlands. A small subset of the participants was recruited via a Facebook advertisement (3.8% of the total sample). More details on Tr&nds and the survey data collection can be found in the supplementary material.

### Measures

#### Cigarettes and alternative tobacco products

For conventional cigarettes, electronic (e-)cigarettes with nicotine, e-cigarettes without nicotine (‘shisha-pen’) and waterpipe, there was a question asking ‘How old were you when you used this substance/device for the first time?’. Answer categories were ‘I never used this substance/device’, ‘11 years or younger’, ‘12 years’, ‘13 years’, ‘14 years’, ‘15 years’, ‘16 years’, ‘17 years’, ‘18 years or older’ for Cohort I, while for the slightly older Cohort II the highest two categories were ‘19 years’ and ‘20 years or older’. Next, adolescents were asked how often they had used each of the alternative tobacco products in the past 4 weeks, with answer categories ‘0’, ‘1’, ‘2’, ‘3’,…, ‘9’, ‘10–19’ and ‘40 +’. For conventional smoking there was an additional question asking ‘Have you ever smoked, even if this was only one cigarette or a few puffs?’ with answer categories ‘I have never smoked’, ‘I have smoked once or twice to try’, ‘I smoke once in a while, but not every day’, ‘I used to smoke but I quit’ and ‘I smoke every day’.

With the above information, variables reflecting *ever use* (0 = *no*, 1 = *yes*) of conventional cigarettes and each of the alternative tobacco products were created. Those saying ‘I never used this substance’ to the first question were classified as never users while those who provided an age at which they used the substance for the first time were classified as ever users. For conventional cigarettes, this variable was cross-checked with the additional question on smoking behaviour (participants who were classified as never users based on the first question but answered they (used to) smoke to the second question, or the other way around, were set to missing). Variables reflecting *past month use* (0 = *no*, 1 = *yes*) of conventional cigarettes and each of the alternative tobacco products were created with a similar approach, contrasting no use in the past 4 weeks (0 times) to use in the past 4 weeks (1 time or more). Finally, a measure of *smoking status* was created. Those who stated to have never smoked cigarettes or only tried once or twice were classified as never smoker, those who smoked but quit were classified as former smoker and those who smoked once in a while or daily were current smokers. For Cohort I, all variables described here were available at both time points (T0 and T1).

When exploring the cross-use of different alternative tobacco products we found clustering such that adolescents who had used one alternative tobacco product, more often than not also used one of the other alternative tobacco products. There were, however, differences in this clustering, depending on the type of alternative tobacco both within and between cohorts (Supplemental Tables 1 and 2). We therefore analyze e-cigarettes with nicotine, e-cigarettes without nicotine and waterpipe separately instead of taking one measure of overall alternative tobacco use.

#### Sociodemographic variables

Sociodemographic variables were sex (0 = boy, 1 = girl), age (continuous variable, categorized into age categories appropriate for each respective cohort namely 11–13, 14–15 and 16–17 years for cohort I and 14–15, 16–17 and 18–21 years for cohort II), ethnicity (including the most common ethnic groups in the Netherlands and based on birth country of the parents; 0 = Netherlands, 1 = Surinam/Aruba/Netherlands Antilles, 2 = Morocco, 3 = Turkey, 4 = Other) and educational attainment (0 = low, 1 = average, 2 = middle and 3 = high for Cohort I and 0 = low/average, 1 = middle and 2 = high for Cohort II). The category ‘low’ refers to schooling for students with learning difficulties and the lowest level of pre-vocational secondary education, ‘average’ refers to the higher levels of pre-vocational secondary education or vocational education, ‘middle’ refers to higher general secondary education or higher professional education and ‘high’ refers to pre-university education or university. Given the low numbers of students classified as ‘low’ in Cohort II, ‘low’ and ‘average’ were merged into one category.

#### Propensity to smoke

In Cohort I only, a composite score of propensity to smoke was computed based on three risk factors for smoking at T0. The first factor, personality, was assessed with the validated ‘Substance Use Risk Profile Scale’ (SURPS) [20]. The SURPS provides sum scores for anxiety sensitivity, hopelessness, sensation seeking and impulsivity. The other two factors, susceptibility to peer pressure and intention to smoke, have also been consistently shown to predict onset of smoking [21]. Intention to smoke was measured by asking adolescents ‘Are you planning to smoke in the coming 6 months?’, with answer categories ranging from 1 ‘Definitely not’ to 7 ‘Definitely yes’, and susceptibility to peer pressure was measured by asking adolescents ‘Imagine that you are with a group of friends who all smoke. They offer you a cigarette, would you take the cigarette and smoke with them?’, with answer categories ranging from 1 ‘Definitely not’ to 7 ‘Definitely yes’. As was done in a study similar to ours [16], we created a composite smoking propensity score by performing a logistic regression analysis and saving the predicted values. In this logistic regression, smoking of conventional cigarettes at T1 (0 = *no*, 1 = *yes*) was the dependent variable and the SURPS personality traits, susceptibility to peer pressure and intention to smoke at T0 were the independent variables.

### Statistical analysis

#### Descriptives and cross-sectional associations

Prevalence rates were assessed in each cohort separately. We report ever use and past month use of conventional cigarettes, e-cigarettes with nicotine, e-cigarettes without nicotine and waterpipe in both cohorts and across sociodemographic variables (sex, age, ethnicity, educational level). For alternative tobacco products we also report the mean number of times used in the past month.

Next, we tested cross-sectional associations between conventional smoking and alternative tobacco use. In a GEE (Generalized Estimation Equation) analysis, correcting for clustering within schools, the dependent variable was ever use (0 = *no*, 1 = *yes*) of either e-cigarettes with nicotine, e-cigarettes without nicotine or waterpipe while the independent variable was ever use of conventional cigarettes (0 = *no*, 1 = *yes*). Covariates were age, sex and educational attainment. Ethnicity was not added as a covariate given the low numbers of adolescents within the different ethnic groups. To check whether ethnicity affected our results, all GEE analyses were repeated in adolescents of Dutch ethnicity only. All analyses were conducted in SPSS Statistical Software.

#### Longitudinal associations

To investigate whether or not the use of alternative tobacco products predicts the use of conventional cigarettes, longitudinal data (T0 and T1) from Cohort I were analyzed. We first selected adolescents who stated to have never smoked conventional cigarettes at T0. Next, we carried out GEE analysis with ever use of conventional cigarettes at T1 (0 = *no*, 1 = *yes*) as the dependent variable, and ever use of either e-cigarettes with nicotine, e-cigarettes without nicotine or waterpipe (0 = *no*, 1 = *yes*) at T0 as the independent variable. Besides age, sex and educational attainment, a composite score of smoking propensity at T0 was added as covariate as well as an interaction term between propensity to smoke and ever use of e-cigarettes with nicotine/e-cigarettes without nicotine/waterpipe. Intervention status (0 = no school policy intervention, 1 = school policy intervention) was corrected for but not reported here (for results on effects of the intervention see [18]).

#### Correction for multiple testing

Given that we perform analyses for three different alternative tobacco products, Bonferonni correction for multiple testing was applied. For Cohort I, three separate cross-sectional regression analyses resulted in a threshold of statistical significance of < 0.017 (0.05/3). For Cohort II the same threshold was adopted given that there were three separate regression analyses in the cross-sectional sample and three in the longitudinal (sub)sample.

## Results

### Descriptive statistics

In Cohort I, e-cigarettes without nicotine were the most popular of the alternative tobacco products, with a prevalence (29.4%) even higher than that of conventional cigarettes (21.7%) (Table 1). In the slightly older Cohort II, waterpipe was the most popular of the alternative tobacco products (45.3%), but ever use prevalence for conventional cigarette smoking was higher (48.6%) (Table 2). Mean number of times used in the past month among recent users was highest for e-cigarettes with nicotine [11.1 (SD = 14.5) in Cohort I and 9.3 (SD = 13.9) in Cohort II] when compared to e-cigarettes without nicotine ]7.9 (SD = 12.0) and 4.8 (SD = 9.5), respectively] and waterpipe [6.8 (SD = 11.1) and 4.1 (SD = 8.8), respectively].

**Table 1:** Descriptive statistics of cigarette, electronic (e-)cigarette with nicotine, e-cigarette without nicotine and waterpipe use in adolescents aged 11–17 years across sociodemographic characteristics—*Cohort I*

	Cigarettes		E-cigarettes with nicotine			E-cigarettes without nicotine			Waterpipe		
	Ever use (% yes)	Past month use (% yes)	Ever use (% yes)	Past month use (% yes)	Freq past month* [M times (SD)]	Ever use (% yes)	Past month use (% yes)	Freq past month* [M times (SD)]	Ever use (% yes)	Past month use (% yes)	Freq past month* [M times (SD)]
Total (n = 6819)	21.7%	11.2%	13.7%	6.7%	11.1 (14.5)	29.4%	13.2%	7.9 (12.0)	22.1%	11.6%	6.8 (11.1)
Sex											
Boy (n = 3533)	23.3%	11.9%	17.3%	8.0%	12.9 (15.8)	35.3%	15.4%	9.0 (13.2)	26.0%	13.7%	8.1 (12.4)
Girl (n = 3286)	19.9%	10.5%	9.9%	5.3%	8.1 (11.3)	23.1%	10.9%	6.4 (9.8)	17.9%	9.3%	4.9 (8.2)
Age											
11–13 years (n = 2705)	10.2%	3.8%	6.7%	3.6%	11.6 (15.0)	21.7%	10.8%	7.2 (11.5)	11.0%	5.4%	5.9 (9.7)
14–15 years (n = 3705)	27.2%	15.1%	17.1%	8.4%	11.0 (14.5)	34.0%	14.8%	8.1 (12.2)	27.5%	14.6%	7.1 (11.4)
16–17 years (n = 407)	48.7%	26.3%	29.0%	11.6%	10.0 (13.5)	38.3%	14.8%	10.0 (12.8)	45.8%	25.5%	7.0 (10.9)
Ethnicity											
Netherlands (n = 5328)	21.2%	10.9%	13.5%	6.4%	10.4 (13.9)	28.6%	12.5%	7.5 (11.5)	20.9%	10.4%	6.0 (10.0)
Surinam/Aruba/Netherlands Antilles (n = 124)	28.2%	20.5%	14.8%	8.7%	11.5 (14.7)	38.3%	18.8%	6.3 (10.9)	28.1%	18.0%	5.2 (8.6)
Morocco (n = 201)	5.0%	2.9%	6.0%	4.6%	11.0 (16.6)	18.6%	10.2%	8.3 (13.8)	14.1%	9.7%	9.7 (14.1)
Turkey (n = 137)	19.7%	7.1%	8.8%	5.1%	19.7 (19.1)	36.3%	17.0%	8.5 (12.9)	37.8%	20.9%	9.1 (14.0)
Other (n = 689)	26.0%	12.7%	15.3%	7.8%	7.9 (12.2)	34.4%	16.9%	7.7 (11.7)	26.3%	16.1%	7.1 (11.0)
Educational level											
Low (n = 2280)	30.9%	17.4%	19.1%	10.9%	10.9 (14.1)	31.7%	15.9%	8.7 (12.3)	25.2%	14.9%	7.2 (11.0)
Average (n = 2132)	22.5%	10.7%	14.3%	6.3%	9.5 (13.4)	33.6%	15.2%	6.9 (11.0)	24.9%	12.8%	5.5 (9.5)
Middle (n = 1174)	11.1%	4.6%	7.3%	2.5%	8.4 (12.2)	25.9%	9.9%	6.3 (10.5)	16.5%	6.3%	5.8 (9.1)
High (n = 1105)	11.6%	6.0%	7.9%	3.2%	20.5 (18.8)	20.4%	7.1%	11.6 (15.5)	14.7%	7.4%	10.8 (15.8)
Ever use cigarettes											
No (n = 5149)	–	–	3.7%	1.5%	7.0 (11.9)	16.9%	6.8%	4.5 (7.9)	10.0%	4.2%	4.2 (8.2)
Yes (n = 1423)	–	–	47.5%	23.1%	11.6 (14.6)	70.9%	33.9%	9.8 (13.3)	62.3%	36.0%	7.3 (11.1)
Cigarette smoking status											
Never smoker (n = 5945)	–	–	7.3%	3.2%	10.1 (14.2)	23.1%	9.7%	6.3 (10.4)	15.0%	7.0%	6.0 (10.5)
Former smoker (n = 146)	–	–	40.1%	14.1%	7.2 (11.5)	60.6%	29.6%	9.7 (13.6)	53.6%	31.7%	6.6 (11.1)
Current smoker (n = 699)	–	–	63.4%	35.5%	12.1 (14.8)	76.4%	40.0%	11.2 (14.1)	75.0%	47.0%	8.0 (11.7)

* Frequency in the past month for those stating that they had used in the past month, the mean was computed from answer categories 0, 1, 2, 3, 4, 5, 6, 7, 8, 9, 10–19 (recoded to 15), 20–39 (recoded to 30) and 40 + (recoded to 40). N.a.: not available because the number of participants was too low (≤ 5). For the variable ethnicity adolescents were classified as one of the non-Dutch categories when one or both of their parents were born in this country. Adolescents whose parents originated from two different countries (both of which were non-Dutch) were excluded from this table (n = 48)

**Table 2:** Descriptive statistics of cigarette, electronic (e-)cigarette with nicotine, e-cigarette without nicotine and waterpipe use in adolescents aged 14–21 years across sociodemographic characteristics—*Cohort II*

	Cigarettes		E-cigarettes with nicotine			E-cigarettes without nicotine			Waterpipe		
	Ever use (% yes)	Past month use (% yes)	Ever use (% yes)	Past month use (% yes)	Freq past month* [M times (SD)]	Ever use (% yes)	Past month use (% yes)	Freq past month* [M times (SD)]	Ever use (% yes)	Past month use (% yes)	Freq past month* [M times (SD)]
Total (n = 2758)	48.6%	24.9%	12.3%	2.5%	9.3 (13.9)	27.6%	2.6%	4.8 (9.5)	45.3%	8.6%	4.1 (8.8)
Sex											
Boy (n = 1066)	46.6%	25.9%	15.2%	3.2%	11.2 (16.3)	36.6%	3.4%	6.8 (12.5)	48.3%	11.8%	4.9 (9.8)
Girl (n = 1692)	49.9%	24.2%	10.5%	2.0%	7.5 (10.9)	22.1%	2.0%	2.7 (3.6)	43.4%	6.6%	3.2 (7.4)
Age											
14–15 years (n = 479)	28.7%	11.9%	7.8%	1.7%	10.8 (15.5)	35.7%	5.7%	3.5 (7.9)	27.8%	8.9%	3.2 (8.0)
16–17 years (n = 1100	43.1%	23.3%	12.5%	3.3%	10.6 (15.3)	33.9%	2.4%	7.2 (12.9)	38.9%	7.6%	4.1 (8.9)
18–21 years (n = 1179)	61.8%	33.3%	13.9%	2.1%	7.1 (11.3)	18.6%	1.4%	3.3 (3.6)	58.3%	9.3%	4.4 (9.0)
Ethnicity											
Netherlands (n = 2246)	47.8%	25.2%	11.1%	2.4%	9.9 (14.3)	26.9%	2.4%	5.3 (10.6)	41.7%	6.9%	3.0 (7.0)
Surinam/Aruba/Netherlands Antilles (n = 52)	58.8%	29.7%	20.0%	4.1%	n.a.	41.7%	6.1%	n.a.	62.0%	16.0%	4.1 (2.5)
Morocco (n = 56)	14.5%	6.0%	7.3%	3.8%	n.a.	9.3%	1.9%	n.a.	27.3%	15.1%	14.6 (16.1)
Turkey (n = 57)	38.6%	8.9%	12.0%	1.9%	n.a.	34.0%	5.7%	n.a.	80.4%	24.5%	8.9 (14.3)
Other (n = 114)	51.9%	31.5%	18.6%	5.4%	n.a.	41.0%	6.5%	1.7 (3.6)	57.7%	22.3%	9.3 (13.3)
Educational level											
Low/average (n = 942)	49.1%	28.5%	14.1%	4.3%	11.7 (15.8)	33.0%	4.6%	5.4 (10.5)	47.9%	14.3%	5.2 (10.0)
Middle (n = 754)	51.0%	28.3%	13.0%	2.8%	5.7 (10.7)	31.7%	2.3%	4.6 (9.8)	44.7%	6.6%	4.5 (9.1)
High (n = 1012)	46.1%	18.5%	10.1%	0.7%	5.0 (5.6)	20.1%	1.0%	2.6 (3.5)	43.8%	5.4%	1.2 (3.1)
Ever use cigarettes											
No (n = 1384)	–	–	1.6%	0.2%	1.3 (1.5)	13.9%	1.2%	2.1 (3.0)	17.8%	3.1%	5.4 (9.7)
Yes (n = 1309)	–	–	24.1%	5.0%	9.9 (14.2)	41.9%	4.1%	5.8 (10.7)	74.0%	14.1%	3.8 (8.6)
Cigarette smoking status											
Never smoker (n = 2025)	–	–	4.8%	0.7%	1.7 (3.2)	20.3%	1.4%	1.7 (2.6)	31.7%	4.8%	4.4 (8.7)
Former smoker (n = 123)	–	–	29.6%	3.3%	n.a.	43.9%	3.3%	n.a.	87.6%	20.8%	3.4 (8.4)
Current smoker (n = 608)	–	–	34.8%	8.4%	11.5 (14.7)	49.4%	6.4%	7.3 (12.1)	82.1%	18.7%	4.0 (9.0)

*Frequency in the past month for those stating that they had used in the past month, the mean was computed from answer categories 0, 1, 2, 3, 4, 5, 6, 7, 8, 9, 10–19 (recoded to 15), 20–39 (recoded to 30) and 40 + (recoded to 40). N.a.: not available because the number of participants was too low (≤ 5). For the variable ethnicity adolescents were classified as one of the non-Dutch categories when one or both of their parents were born in this country. Adolescents whose parents originated from two different countries (both of which were non-Dutch) were excluded from this table (n = 7)

For conventional smoking, sex differences were small, with slightly more boys than girls having ever smoked in Cohort I and a higher prevalence in girls compared to boys in Cohort II. In contrast, alternative tobacco (ever and past month) use was markedly higher in boys compared to girls in both cohorts. In Cohort I, conventional smoking and alternative tobacco use was more prevalent in the older age groups. In Cohort II a similar trend was seen except for electronic cigarettes without nicotine, which were less popular in the older age groups. Ever using cigarettes and alternative tobacco products was more prevalent among adolescents belonging to the ethnic group ‘Surinam/Aruba/Netherlands Antilles’ than adolescents whose parents originated from the Netherlands. Adolescents of Moroccan descent had used conventional cigarettes or alternative tobacco products less often compared to all other groups. Among adolescents of Turkish descent use of e-cigarettes was just as common as it was among adolescents with parents born in the Netherlands, while the prevalence of waterpipe was higher. Finally, a higher educational level was generally associated with a lower use of cigarettes and alternative tobacco products in both cohorts.

Although alternative tobacco use was most common among adolescents who smoked conventional cigarettes before, there were adolescents who never tried smoking a conventional cigarette (not even a few puffs) but who had tried an alternative tobacco product (ranging from 1.6 to 17.8% across cohorts and type of alternative tobacco product).

### Cross-sectional associations

Ever having used a conventional cigarette was strongly associated with ever use of e-cigarettes with nicotine [OR 20.04 (95% CI 14.84–27.06) in Cohort I, OR 19.70 (CI 13.81–28.09) in Cohort II] e-cigarettes without nicotine [13.17 (CI 10.77–16.10), 7.31 (CI 5.34–10.03), respectively] and waterpipe [13.76 (CI 11.48–16.49), 11.86 (CI 9.26–15.20), respectively] (Tables 3, 4). From these GEE models we can also derive the effects of sex, age and education on alternative tobacco use, when corrected for each other and for conventional smoking. For all alternative tobacco products and in both cohorts, there was strong evidence for girls being at lower odds of ever use than boys. In Cohort I an increasing age was associated with an increased odds of ever using e-cigarettes with nicotine and waterpipe, while for e-cigarettes without nicotine there was no clear pattern. In Cohort II there was no clear pattern of age on e-cigarettes with nicotine while the use of e-cigarettes without nicotine was markedly lower in the older age groups and the use of waterpipe was higher in older age groups. There was no clear evidence for an association between educational level and alternative tobacco use. Results were similar when repeating analyses only in individuals with both parents born in the Netherlands (data not shown).

**Table 3:** Generalized Estimating Equation (GEE) analyses with ever use of electronic (e-)cigarettes with nicotine/e-cigarettes without nicotine/waterpipe as the dependent variable and ever use of conventional cigarettes as the independent variable—*Cohort I*

	Ever use e-cigarettes with nicotine (n = 6268)			Ever use e-cigarettes without nicotine (n = 6260)			Ever use waterpipe (n = 6263)		
	OR	95% CI	*p* value	OR	95% CI	*p* value	OR	95% CI	*p* value
Ever use cigarettes									
No	1.00	–	–	1.00	–	–	1.00	–	–
Yes	20.04	14.84–27.06	< 0.001	13.17	10.77–16.10	< 0.001	13.76	11.48–16.49	< 0.001
Sex									
Boy	1.00	–	–	1.00	–	–	1.00	–	–
Girl	0.52	0.43–0.64	< 0.001	0.51	0.42–0.63	< 0.001	0.63	0.53–0.76	< 0.001
Age									
11–13 years	1.00	–	–	1.00	–	–	1.00	–	–
14–15 years	1.61	1.20–2.15	0.001	1.23	1.01–1.49	0.039	2.14	1.83–2.49	< 0.001
16–17 years	1.90	1.20–3.00	0.006	0.79	0.63–0.98	0.031	3.42	2.75–4.27	< 0.001
Educational level									
Low	1.00	–	–	1.00	–	–	1.00	–	–
Average	0.86	0.59–1.24	0.416	1.58	1.05–2.39	0.030	1.57	1.02–2.42	0.041
Middle	0.65	0.42–0.99	0.043	1.52	1.14–2.03	0.005	1.42	1.05–1.92	0.022
High	0.70	0.43–1.15	0.163	1.02	0.73–1.43	0.901	1.17	0.77–1.78	0.462

Bonferonni corrected *p* value level of significance was 0.017. For Cohort I, GEE analyses were additionally corrected for intervention status (see [18])

**Table 4:** Generalized Estimating Equation (GEE) analyses with ever use of electronic (e-)cigarettes with nicotine/e-cigarettes without nicotine/waterpipe as the dependent variable and ever use of conventional cigarettes as the independent variable—*Cohort II*

	Ever use e-cigarettes with nicotine (n = 2544)			Ever use e-cigarettes without nicotine (n = 2526)			Ever use waterpipe (n = 2584)		
	OR	95% CI	*p* value	OR	95% CI	*p* value	OR	95% CI	*p* value
Ever use cigarettes									
No	1.00	–	–	1.00	–	–	1.00	–	–
Yes	19.70	13.81–28.09	< 0.001	7.45	5.44–10.21	< 0.001	11.92	9.28–15.31	< 0.001
Sex									
Boy	1.00	–	–	1.00	–	–	1.00	–	–
Girl	0.65	0.44–0.94	0.025	0.53	0.41–0.67	< 0.001	0.61	0.43–0.88	0.007
Age									
14–16 years	1.00	–	–	1.00	–	–	1.00	–	–
17–18 years	1.35	1.05–1.74	0.021	0.66	0.48–0.90	0.009	1.46	0.99–2.15	0.055
19–21 years	1.07	0.76–1.50	0.719	0.20	0.14–0.28	< 0.001	2.71	1.90–3.87	< 0.001
Educational level									
Low/average	1.00	–	–	1.00	–	–	1.00	–	–
Middle	0.90	0.56–1.42	0.636	1.00	0.72–1.38	0.982	0.76	0.47–1.24	0.274
High	0.78	0.45–1.36	0.381	0.80	0.59–1.10	0.163	0.72	0.47–1.10	0.126

Bonferonni corrected *p* value level of significance was 0.017

### Longitudinal associations

In adolescents who had never smoked a conventional cigarette at T0, ever use of alternative tobacco products was associated with a higher odds of conventional smoking at T1 (see Table 5). That is, adolescents who ever used an e-cigarette with nicotine were at 11.90 higher odds of having smoked a conventional cigarette 6 months later, than those who never used an e-cigarette with nicotine (95% CI 3.36–42.11). These odds were 5.36 (95% CI 2.73–10.52) for e-cigarettes without nicotine and 5.36 (95% CI 2.78–10.31) for waterpipe.

**Table 5:** Longitudinal Generalized Estimating Equation (GEE) analyses with ever use of conventional cigarettes at T1 as the dependent variable and ever use of electronic (e-)cigarettes with nicotine/e-cigarettes without nicotine/waterpipe at T0 as the independent variable *in adolescents who never smoked a conventional cigarette at T*0—*Cohort I*

	Ever use cigarettes T1 (n = 2100)			Ever use cigarettes T1 (n = 2099)			Ever use cigarettes T1 (n = 2100)		
	OR	95% CI	*p* value	OR	95% CI	*p* value	OR	95% CI	*p* value
Ever use alternative tobacco product T0	E-cigarettes with nicotine			E-cigarettes without nicotine			Waterpipe		
No	1.00	–	–	1.00	–	–	1.00	–	–
Yes	11.90	3.36–42.11	< 0.001	5.36	2.73–10.52	< 0.001	5.36	2.78–10.31	< 0.001
Sex									
Boy	1.00	–	–	1.00	–	–	1.00	–	–
Girl	1.25	0.87–1.80	0.223	1.40	0.95–2.07	0.088	1.26	0.87–1.81	0.217
Age									
11–13 years	1.00	–	–	1.00	–	–	1.00	–	–
14–15 years	1.55	1.06–2.28	0.025	1.56	1.07–2.29	0.022	1.51	1.04–2.18	0.029
16–17 years	1.38	0.30–6.46	0.681	1.67	0.36–7.73	0.510	1.22	0.29–5.05	0.789
Educational level									
Low	1.00	–	–	1.00	–	–	1.00	–	–
Average	1.01	0.66–1.52	0.981	0.93	0.57–1.51	0.763	1.03	0.68–1.57	0.874
Middle	0.66	0.37–1.16	0.151	0.56	0.29–1.09	0.088	0.65	0.35–1.20	0.170
High	0.43	0.20–0.93	0.033	0.39	0.17–0.88	0.023	0.42	0.18–0.90	0.026
Propensity to smoke									
SD increase	68.21	24.24–192.00	< 0.001	56.57	15.93–200.91	< 0.001	73.79	21.28–255.96	< 0.001
Interaction term									
SD increase	0.02	0.00–0.37	0.016	0.18	0.02–1.82	0.147	0.05	0.01–0.49	0.010

Bonferonni corrected *p* value level of significance was 0.017. For Cohort I, GEE analyses were additionally corrected for intervention status (see [18]). Propensity to smoke represents a composite score based on personality, susceptibility to peer pressure and intention to smoke, while the interaction term represents an interaction between propensity to smoke and ever use of the alternative tobacco product in question

The composite score of propensity to smoke at T0—reflecting personality traits strongly correlated with substance use, susceptibility to peer pressure and intention to smoke—was a strong predictor of smoking conventional cigarettes at T1 (ORs ranging between 56.57 and 73.79, *p* < 0.001). Interestingly, there was strong evidence for an interaction between propensity to smoke and alternative tobacco use at baseline. ORs for the interaction terms between propensity to smoke and e-cigarette with nicotine use at baseline and between propensity to smoke and waterpipe use at baseline were 0.02 (95% CI 0.00–0.37) and 0.05 (95% CI 0.01–0.49), respectively. This indicates that the association between alternative tobacco use at T0 and smoking T1 was *weaker* for individuals who had a strong propensity to smoke in the first place (a more ‘at risk’ personality, higher susceptibility to peer pressure and higher intention to smoke). Thus, there was a *stronger* association for those who have a low propensity to smoke at baseline. For e-cigarettes without nicotine there was similar, but weaker, evidence with the interaction term showing a similar direction of effect but not reaching significance. When performing a median split on propensity to smoke and repeating GEE analyses, we found that alternative tobacco use at T0 predicted conventional smoking at T1 in both groups, but the association was much stronger for the low propensity scorers (ORs for e-cigarettes with nicotine, e-cigarettes without nicotine and waterpipe were 7.80, 6.07 and 4.22, respectively) than for the high propensity scorers (ORs were 2.89, 3.30 and 2.57, respectively). See Supplemental Tables 3 and 4. Results were similar when only selecting adolescents with both parents born in the Netherlands (data not shown).

Of the adolescents who had never smoked conventional cigarettes at T0 but who initiated smoking at T1 after they used an alternative tobacco product, the majority stated that they only smoked once or twice in their lifetime (77.9%) and when asked about recent smoking behaviour only 25.3% said they smoked in the past month.

## Discussion

In two large representative cohorts of Dutch adolescents, experimenting with alternative tobacco products (e-cigarettes with nicotine, e-cigarettes without nicotine and waterpipe) was popular, while recent or regular use was less common. We showed that among adolescents who never smoked at baseline, experimentation with alternative tobacco products was associated with a higher risk of conventional smoking 6 months later. Importantly, this association was especially strong for adolescents who were initially at low risk of smoking as based on personality, susceptibility to peer pressure and intention to smoke. We are the first to report these longitudinal findings for e-cigarettes with nicotine and without nicotine separately, as well as for waterpipe.

In the present study we found that 13.7% of a cohort of 11–17 year old adolescents ever used an e-cigarette with nicotine while 29.4% ever used an e-cigarette without nicotine. For 14–21 year olds this was 12.3 and 27.6%, respectively. These prevalence rates are very comparable to previous research in Dutch adolescents [12]. Ever use of waterpipe (22.1%) was also similar to previous findings for the 11–17 year old cohort [12] while for 14–21 year olds we found a markedly higher prevalence of 45.3%. Combined with the fact that within both cohorts the higher age groups showed the highest waterpipe use rates, this suggests that this behaviour is more popular among young adults than among adolescents. For e-cigarettes with nicotine a higher age was also associated with a higher prevalence of use within both cohorts. The popularity of e-cigarettes without nicotine was especially low in the highest age groups (17–18 and 19–21 years). This may be due to the fact that e-cigarettes without nicotine, also called shisha-pens, are produced in different colours and flavours (such as cola, cherry or peach) [5] which make them particularly attractive for younger adolescents. For all alternative tobacco products and in both cohorts, prevalence rates were lower in girls, again comparable to earlier findings [12].

Our finding that the use of alternative tobacco products was strongly associated with smoking conventional cigarettes corroborates previous literature [6, 7, 12, 22–27]. Interestingly, the association between e-cigarettes with nicotine and smoking was stronger than between e-cigarettes without nicotine and smoking. This may have to do with the nicotine content. Not many previous studies have made the distinction we did, while nicotine content is thought to play a major role in use patterns of alternative tobacco [28]. Our findings support evidence suggesting that early exposure to nicotine through routes other than smoking may lead adolescents to smoke conventional cigarettes because they are ‘hooked’ on the nicotine in e-cigarettes and cigarettes deliver nicotine faster [29, 30]. Under this hypothesis, a stronger association for e-cigarettes with than those without nicotine would be expected. In general, we report effect sizes that are higher than what has been reported in the literature, especially for e-cigarettes with nicotine. Since most other studies didn’t distinguish e-cigarettes with nicotine from those without, it may be that previous effect sizes were somewhat dampened. Another explanation could be that there are differences in smoking rates between our Dutch sample and the previous studies which were mostly US-based—smoking prevalence is considerably lower in the US than in most European countries [31].

In never smoking adolescents, alternative tobacco use at baseline was associated with conventional smoking at follow-up, 6 months later. Again, the strongest association was found for e-cigarettes with nicotine. As done earlier by others [16] we computed composite risk scores based on factors known to be predictive of future smoking behaviour. We found a negative interaction such that the link between alternative tobacco products at baseline and conventional smoking 6 months later was stronger for adolescents who were at lower baseline risk of smoking than for adolescents who were at higher risk of smoking. While some recent studies have shown the same effect for e-cigarettes [16, 17], these did not distinguish e-cigarettes *with* nicotine from those *without*. Combined, previous findings and our own suggest that adolescents who were initially at low risk of smoking may have a higher odds of initiating conventional smoking due to having experimented with e-cigarettes. We found similar results for waterpipe use, which predicted conventional smoking. This is in line with the few longitudinal studies published on so far [32, 33] and with a cross-sectional study demonstrating that waterpipe smoking was associated with susceptibility to cigarette smoking [34]. We now show for the first time that the association between baseline waterpipe use and conventional smoking 6 months later is especially strong for adolescents who initially had a low risk of smoking.

Strengths of the present study are its use of two large, representative samples of adolescents and young adults, the distinction between e-cigarettes with and without nicotine, the inclusion of waterpipe use and longitudinal analyses incorporating baseline susceptibility to conventional smoking. As was pointed out in a recent commentary, it remains difficult to definitively test whether alternative tobacco products directly lead to conventional smoking and we need to be careful in labelling alternative tobacco products a ‘gateway’ to conventional smoking [35]. It was also suggested, however, that certain types of studies are especially useful to assess causality. These include large longitudinal epidemiological studies which (precisely) measure smoking onset and confounders and studies that include a propensity score measure of liability to smoking [35]. We incorporated both of these aspects in our study, thereby increasing the strength of our findings. There are also some limitations to consider. In Cohort II adolescents of an ethnicity other than Dutch were slightly underrepresented, making it difficult to draw strong conclusions from the patterns of use among different ethnic groups. Also in Cohort II, girls were slightly overrepresented (61.3% of the total sample). Finally, there may have been selection bias such that our samples were not completely representative of the average Dutch youth. While participants from Cohort I attended schools across the Netherlands, schools included in Cohort II were located mostly in the West of the Netherlands. Overall, however, our findings were very similar to earlier findings in a national Dutch surveillance study [12]. It also needs to be noted that we measured smoking behaviour and alternative tobacco use with surveys (self-report), which may have introduced bias due to over and underreporting [36]. In our longitudinal analyses we applied a follow-up time of 6 months, but a longer follow-up would be needed to better determine the effects of alternative tobacco use on conventional smoking behaviour. Similar to others [15], we found that most of the adolescents who initiated smoking after having first used alternative tobacco products, said that so far they only smoked once or twice. It is unclear whether these low levels of smoking eventually lead to regular cigarette use or not.

In conclusion, our findings clearly show that the use of alternative tobacco products is becoming an increasingly popular (risk) behaviour among youth, and, in line with other recent studies, that the use of these products is associated with (later) smoking of conventional cigarettes. Although we found the strongest effects for e-cigarettes with nicotine, we report similar findings for e-cigarettes without nicotine and waterpipe. Importantly, the link between alternative tobacco use and conventional smoking was strongest among adolescents with a low smoking propensity, which seems to be in line with a ‘gateway’ effect. However, given that it is still largely unclear through which mechanism alternative tobacco products might lead to conventional smoking, we need to be careful with claiming causality. More research is needed, most notably large-scale longitudinal studies that assess the use of different types of alternative tobacco products (both with and without nicotine) and with multiple follow-up measures of (regular) smoking over a longer period of time. As of May 2016, the Dutch government has issued an age limit of 18 years for the use of e-cigarettes [37]. Since most of the data we base our analyses on were collected before or just after that date, it will also be important for future studies to monitor adolescents’ use of e-cigarettes and whether or not this will decline.

## Electronic supplementary material

Below is the link to the electronic supplementary material.

Supplementary material 1 (DOCX 34 kb)
