# Supplementary material for: E-cigarette and waterpipe use in two adolescent cohorts: cross-sectional and longitudinal associations with conventional cigarette smoking
Source: Eur J Epidemiol. 2017 Dec 19;33(3):323–34. doi: 10.1007/s10654-017-0345-9 (PMC5889768; doi:10.1007/s10654-017-0345-9)
Supplement: Supplementary file 1 — Supplementary material 1 (DOCX 34 kb) [file 10654_2017_345_MOESM1_ESM.docx]

**Supplementary material**

*‘E-cigarette and waterpipe use in two adolescent cohorts: cross-sectional and longitudinal associations with conventional cigarette smoking’*

Jorien L. Treur, Andrea D. Rozema, Jolanda J.P. Mathijssen, Hans van Oers & Jacqueline M. Vink

**Content:**

Page 2-4 Description of Cohort I and Cohort II

Page 5 Supplementary Table 1 and Table 2. Cross-use of electronic cigarettes with nicotine, electronic cigarettes without nicotine and waterpipe

Page 6-7 Supplemental Table 3 and Table 4. Longitudinal Generalized Estimating Equation (GEE) analyses

**Description of the study cohorts**

**Cohort I**

Cohort I consisted of participants that took part in a study aimed at investigating the effects of school smoking policy on the smoking behaviour of students. The study employed a quasi-experimental design, such that schools that were planning to implement an outdoor school ground smoking ban were compared to schools that had no intention to implement such a policy. First, the schools’ current smoking policy, and future intentions to implement an outdoor smoking ban, was identified in a national monitor questionnaire for 919 Dutch secondary schools spread across the Netherlands [23]. From this list, a total of 77 schools were randomly selected, contacted by telephone and asked whether they would participate in the study. Of these 77 school, 19 were willing to participate. Initially, the ‘experimental’ condition consisted of 9 schools that were intending to implement an outdoor school ground smoking ban and the ‘control’ condition consisted of 10 schools that had no such intention. Data were collected in 2014 and 2015. There was a baseline measurement (T0) and a follow-up measurement approximately 6 months later (T1). The first survey (T0) was completed on average two months before implementation and the second (T1) four months after implementation of the outdoor smoking ban. During the course of the baseline measurements, one school in the control condition dropped out, as this school wanted to implement an outdoor smoking ban. During analysis the available data at T0 of this school was used and we corrected for school smoking policy. Both surveys included a wide range of questions on smoking behaviour and alternative tobacco use, as well as questions on personality traits related to substance use and demographic characteristics. Surveys were administered in classrooms (either online in a computer room or on paper) under the supervision of a teacher.

The Psychological Ethics Committee of Tilburg University approved the study (EC-2014.19) and all participants provided informed consent. Parents of all students were informed about the research beforehand and informed that they could indicate if they did not want their child to participate (passive consent). A small minority (*n*=30) refused study participation of their child so these students did not participate in the study. On an individual-level (vs. school-level), 2,982 adolescents (who all gave informed consent) had data available from at least one time-point (T0 and/or T1). Individuals were excluded in the following situations; when information on the school they attended was missing (n=54), when they were aged <11 years or >17 years or when information on age or gender was missing (n=109). This resulted in a study sample of n=6,819 (mean age=13.8 (SD=1.1), 48.2% female). Of these, 2,100 adolescents had longitudinal data available (T0 and T1).

**Cohort II - Tr&nds study (traditional & novel substance use among adolescents)**

Tr&nds aims to assess a wide range of addictive behaviours among Dutch adolescents. The study was conducted in 2016 and 2017, when surveys were administered at secondary schools and at the three main levels of follow-up schooling that exist in the Netherlands: vocational education, higher professional education and university. In total, 14 educational institutions participated in the study, mostly located in the west of the Netherlands. In addition, a small subset of the participants was recruited via a Facebook advertisement (3.8% of the total sample). The surveys included questions on different addictive behaviours that are relevant to this age group; smoking behaviour, alcohol and cannabis use, use of alternative tobacco products (electronic cigarettes, shisha pen [electronic cigarette without nicotine] and waterpipe), food addiction (Yale Food Addiction Scale for Children), sugar intake through drinks, as well as questions on personality traits related to substance use, and sociodemographic factors. The surveys were administered in the classroom in the case of secondary schools and vocational education institutions, either on paper or online in computer rooms. If the participants were students of higher professional education institutions or universities, they were invited to complete the survey online in their own time.

Ethical approval for Tr&nds was obtained from the Ethics Committee of Social Sciences at Radboud University, Nijmegen (ECSW2016-1403-381) and all participants provided informed consent. When participants where younger than 16 years, their parents were informed beforehand about the study and were informed that they could indicate if they did not want their child to participate (passive consent). In total, 2885 adolescents were initially enrolled. Individuals were excluded in the following situations; when informed consent was missing or incomplete (n=42), when they were aged >21 years or when information on age or gender was missing (n=85). This resulted in a final study sample of n=2,758 (mean age=17.3, SD=1.8, 61.3% female).

**Supplementary Table 1.** Cross-use of electronic cigarettes with nicotine, electronic cigarettes without nicotine and waterpipe – *Cohort I*

|  |  | Ever use e-cigarette with nicotine | | Ever use e-cigarette without nicotine | |
| --- | --- | --- | --- | --- | --- |
|  |  | Yes | No | Yes | No |
| Ever use e-cigarette without nicotine | Yes | 677 (74.8%) | 779 (13.7%) | - | - |
|  | No | 228 (25.2%) | 4,926 (86.3%) | - | - |
|  | *Total* | *905 (100%)* | *5,705 (100%)* | *-* | - |
| Ever use waterpipe | Yes | 772 (85.1%) | 1,169 (20.5%) | 1,145 (58.9%) | 310 (6.6%) |
|  | No | 135 (14.9%) | 4,533 (79.5%) | 798 (41.1%) | 4,353 (93.4%) |
|  | *Total* | *907 (100%)* | *5,702 (100%)* | *1,943 (100%)* | *4,663 (100%)* |

**Supplementary Table 2.** Cross-use of electronic cigarettes with nicotine, electronic cigarettes without nicotine and waterpipe – *Cohort II*

|  |  | Ever use e-cigarette with nicotine | | Ever use e-cigarette without nicotine | |
| --- | --- | --- | --- | --- | --- |
|  |  | Yes | No | Yes | No |
| Ever use e-cigarette without nicotine | Yes | 210 (69.5%) | 477 (21.1%) | - | - |
|  | No | 92 (30.5%) | 1,780 (78.9%) | - | - |
|  | *Total* | *302 (100%)* | *2,257 (100%)* | - | - |
| Ever use waterpipe | Yes | 273 (86.1%) | 875 (38.3%) | 532 (75.0%) | 609 (32.4%) |
|  | No | 44 (13.9%) | 1,412 (61.7%) | 177 (25.0%) | 1,269 (67.6%) |
|  | *Total* | *317 (100%)* | *2,287 (100%)* | *709 (100%)* | *1,878 (100%)* |

**Supplementary Table 3.** Longitudinal Generalized Estimating Equation (GEE) analyses with ever use of conventional cigarettes at T1 as the dependent variable and ever use of electronic (e-)cigarettes with nicotine / e-cigarettes without nicotine / waterpipe at T0 as the independent variable, corrected for clustering within schools and covariates sex, age and educational level, *in adolescents who never smoked a conventional cigarette at T0* *and with a below median propensity of conventional smoking at T0* – *Cohort I*

|  | *Ever use cigarettes T1*  *(n=1,050)* | | | *Ever use cigarettes T1*  *(n=1,049)* | | | *Ever use cigarettes T1*  *(n=2,100)* | | |
| --- | --- | --- | --- | --- | --- | --- | --- | --- | --- |
|  | **OR** | **95% CI** | **p-value** | **OR** | **95% CI** | **p-value** | **OR** | **95% CI** | **p-value** |
| *Ever use alternative tobacco product T0* | *E-cigarettes with nicotine* | |  | *E-cigarettes without nicotine* | | | *Waterpipe* |  |  |
| No | 1.00 | - | - | 1.00 | - | - | 1.00 | - | - |
| Yes | 7.80 | 1.90 – 32.04 | 0.004 | 6.07 | 2.18 – 16.90 | 0.001 | 4.22 | 1.95 – 9.17 | <0.001 |
| *Sex* |  |  |  |  |  |  |  |  |  |
| Boy | 1.00 | - | - | 1.00 | - | - | 1.00 | - | - |
| Girl | 2.29 | 1.12 – 4.78 | 0.023 | 2.68 | 1.30 – 5.51 | 0.008 | 2.35 | 1.16 – 4.74 | 0.018 |
| *Age* |  |  |  |  |  |  |  |  |  |
| 11-13 years | 1.00 | - | - | 1.00 | - | - | 1.00 | - | - |
| 14-15 years | 2.41 | 1.17 – 4.96 | 0.017 | 2.40 | 1.18 – 4.89 | 0.016 | 2.38 | 1.18 – 4.80 | 0.016 |
| 16-17 years | 1.70 | 0.18 – 15.77 | 0.642 | 1.93 | 0.21 – 17.64 | 0.561 | 1.49 | 0.24 – 9.23 | 0.670 |
| *Educational level* |  |  |  |  |  |  |  |  |  |
| Low | 1.00 | - | - | 1.00 | - | - | 1.00 | - | - |
| Average | 1.00 | 0.45 – 2.25 | 0.994 | 0.89 | 0.34 – 2.33 | 0.816 | 1.10 | 0.50 – 2.41 | 0.812 |
| Middle | 0.36 | 0.13 – 1.00 | 0.049 | 0.31 | 0.11 – 0.89 | 0.030 | 0.36 | 0.13 – 0.96 | 0.042 |
| High | 0.38 | 0.13 – 1.08 | 0.070 | 0.37 | 0.13 – 1.04 | 0.059 | 0.38 | 0.14 – 1.05 | 0.062 |

Note: Bonferroni corrected p-value level of significance was 0.017. Analyses were additionally corrected for intervention status (see Rozema et al., 2017).

**Supplementary Table 4.** Longitudinal Generalized Estimating Equation (GEE) analyses with ever use of conventional cigarettes at T1 as the dependent variable and ever use of electronic (e-)cigarettes with nicotine / e-cigarettes without nicotine / waterpipe at T0 as the independent variable, corrected for clustering within schools and covariates sex, age and educational level, *in adolescents who never smoked a conventional cigarette at T0 and with an above median propensity of conventional smoking at T0* – *Cohort I*

|  | *Ever use cigarettes T1*  *(n=1,050)* | | | *Ever use cigarettes T1*  *(n=1,050)* | | | *Ever use cigarettes T1*  *(n=1,050)* | | |
| --- | --- | --- | --- | --- | --- | --- | --- | --- | --- |
|  | **OR** | **95% CI** | **p-value** | **OR** | **95% CI** | **p-value** | **OR** | **95% CI** | **p-value** |
| *Ever use alternative tobacco product T0* | *E-cigarettes with nicotine* | |  | *E-cigarettes without nicotine* | |  | *Waterpipe* |  |  |
| No | 1.00 | - | - | 1.00 | - | - | 1.00 | - | - |
| Yes | 2.89 | 1.47 – 5.68 | 0.002 | 3.30 | 2.33 – 4.67 | <0.001 | 2.57 | 1.70 – 3.89 | <0.001 |
| *Sex* |  |  |  |  |  |  |  |  |  |
| Boy | 1.00 | - | - | 1.00 | - | - | 1.00 | - | - |
| Girl | 1.08 | 0.75 – 1.56 | 0.664 | 1.18 | 0.79 – 1.78 | 0.424 | 1.10 | 0.75 – 1.61 | 0.618 |
| *Age* |  |  |  |  |  |  |  |  |  |
| 11-13 years | 1.00 | - | - | 1.00 | - | - | 1.00 | - | - |
| 14-15 years | 1.31 | 0.81 – 2.12 | 0.025 | 1.34 | 0.84 – 2.14 | 0.225 | 1.26 | 0.78 – 2.03 | 0.344 |
| 16-17 years | 1.39 | 0.25 – 7.84 | 0.711 | 1.66 | 0.28 – 9.77 | 0.574 | 1.22 | 0.21 – 7.24 | 0.827 |
| *Educational level* |  |  |  |  |  |  |  |  |  |
| Low | 1.00 | - | - | 1.00 | - | - | 1.00 | - | - |
| Average | 1.09 | 0.68 – 1.75 | 0.708 | 0.97 | 0.57 – 1.65 | 0.897 | 1.05 | 0.66 – 1.68 | 0.827 |
| Middle | 0.91 | 0.54 – 1.53 | 0.707 | 0.77 | 0.43 – 1.40 | 0.396 | 0.90 | 0.52 – 1.56 | 0.699 |
| High | 0.58 | 0.19 – 1.73 | 0.328 | 0.47 | 0.15 – 1.46 | 0.192 | 0.56 | 0.18 – 1.73 | 0.313 |

Note: Bonferroni corrected p-value level of significance was 0.017. Analyses were additionally corrected for intervention status (see Rozema et al., 2017).
